# Local Trade‐Offs Shape Flower Size Evolution Across *Arabidopsis thaliana* Distribution

**DOI:** 10.1111/mec.70525

**Published:** 2026-08-28

**Authors:** Kevin Sartori, Clàudia F. Mestre, Md Jonaid Hossain, Aurélien Estarague, Elza Gaignon, Pierre Moulin, Jesse R. Lasky, Denis Vile, Francois Vasseur, Cyrille Violle, Adrien Sicard

**Affiliations:** ^1^ Department of Plant Biology Swedish University of Agricultural, Sciences and Linnean Center for Plant Biology Uppsala Sweden; ^2^ CEFE, Univ Montpellier, CNRS, EPHE, IRD Montpellier France; ^3^ Department of Biology and PAC Herbarium Pennsylvania State University University Park Pennsylvania USA; ^4^ Laboratoire D'ecophysiologie Des Plantes Sous Stress Environnementaux (LEPSE), INRA, Montpellier SupAgro, UMR Montpellier France

**Keywords:** *Arabidopsis thaliana*, environmental constraints, flower size evolution, phenotypic diversity

## Abstract

The remarkable variation in floral form and size is thought to have driven the evolutionary radiation of angiosperms and has traditionally been attributed to pollinator‐mediated selection on floral displays, yet emerging evidence indicates that abiotic environmental factors can also generate and filter variation in flower size. Here, we investigate the geographic variation in reproductive organ growth in the self‐fertilising species *Arabidopsis thaliana* , where the loss of pollinator dependence is predicted to favour reduced floral investment through resource reallocation. We find extensive variation in flower size underpinned by a polygenic architecture, in which derived alleles both increase or decrease petal size and show signatures of positive selection. Nevertheless, the evolutionary direction of flower size differs across geographic regions, reflecting environmentally mediated shifts in the relationship between flower size and seed production. Purifying selection favours small flowers at the climatic margins of the species, but is relaxed in more suitable habitats allowing the emergence and persistence of both small and large‐flower variants. This biogeographic pattern extends to other life‐history traits, highlighting how environmental heterogeneity and constraints shape evolutionary trajectories and maintain phenotypic diversity in selfing lineages.

## 1 Introduction

Natural selection balances flower organ construction and maintenance costs against the likelihood of fertilisation (Roddy, Martínez‐Perez, et al. 2020). These costs follow classical allocation theory, as illustrated by the trade‐off between flower size and number (Caruso 2006; Roddy, Théroux‐Rancourt, et al. 2020; Worley and Barrett 2000). In animal‐pollinated outcrossing species, pollinator‐mediated selection favours large, nutritious and conspicuous flowers to meet pollinator nutritional needs and behaviours (Crane et al. 1994; Friis et al. 1994, 1999, 2006; Galen 1999; Gervasi and Schiestl 2017; Parachnowitsch and Kessler 2010). Following the transitions to self‐fertilisation, however, natural selection is expected to reduce investment in floral displays and attractiveness for the benefit of other fitness components (Stebbins 1957). For instance, seed size and number, two major components of plant life‐history strategies (Salguero‐Gómez 2017), are tightly associated with flower size across flowering plants (Bawa et al. 2019). These relationships are frequently invoked to explain the characteristic reduction in flower size and attractiveness, collectively known as the ‘selfing syndrome’, observed in selfing species (Sicard et al. 2011). Yet, empirical studies quantifying the relative importance of resource‐allocation trade‐offs compared to other evolutionary processes remain limited.

Directional selection and genetic drift can both influence floral evolution in self‐fertilising lineages, each leading to distinct patterns of flower size variation. When the construction costs of floral organs are significant, selection should favour mutations that reduce flower size, thereby enhancing seed production through resource reallocation (Bennett et al. 2012; Charlesworth and Charlesworth 1981). This directional selection scenario predicts (i) a negative association between flower size and seed production, (ii) genomic evidence of positive selection on alleles that reduce flower size and (iii) a general convergence towards smaller flowers with reduced within‐species variance (Stephan 2019). Because environmental heterogeneity influences both resource availability and selection strength (Troth et al. 2018), these patterns should be more pronounced in less favourable environments (Fu et al. 2010; MacTavish and Anderson 2020; Tuller et al. 2018). In contrast, when the construction costs of flower organs are low, the loss of attractive traits could confer little to no fitness benefit and selection maintaining large flowers would be relaxed. Under this scenario, the trajectory of floral‐size evolution may become less predictable, with genetic drift playing a more dominant role, widening phenotypic variance (Buysse et al. 2025) and weakening signatures of directional selection at causal loci (Woźniak and Sicard 2018). Reduced effective recombination under high homozygosity can further facilitate drift‐mediated fixation of nonsynonymous mutations that impair floral‐growth genes (Barrett et al. 2014). Vegetative organs such as leaves are subject to different functional constraints and selective regimes than flowers, so their size is expected to follow distinct evolutionary trajectories that provide a useful contrast for testing predictions on flower‐size evolution.

Additional processes may interact with these trajectories. Pleiotropy can couple floral size variation with other organs controlled by shared developmental pathways, particularly for ontogenically related structures such as floral organs and leaves. The resulting covariation can range from near isometric to clearly allometric, depending on the extent of shared genetic control (Huxley et al. 1993; Johnson and Lenhard 2011). Likewise, linked selection, enhanced in selfers due to low effective recombination, can further reshape diversity around selected loci and indirectly hitchhike alleles affecting floral size (Slotte 2014). Given the diversity and nature of the mechanisms involved, floral‐size variation is likely to be highly polygenic, consistent with observations in selfing lineages (Abraham et al. 2013; Sicard et al. 2011; Sicard and Lenhard 2011; Woźniak et al. 2020). However, the relative contributions of these mechanisms remain poorly understood.

*Arabidopsis thaliana* provides a powerful system to test these predictions. The evolution of self‐fertilisation in this species occurred relatively recently and was followed by rapid range expansion across diverse climates and environments (Bechsgaard et al. 2006; Shimizu and Tsuchimatsu 2015; Tang et al. 2007). This history provides opportunities for environment‐specific selection on floral investment and the fixation of distinct size‐affecting mutations across lineages (Tsuchimatsu et al. 2020). It thus allows us to test the relative importance of resource allocation and environmental heterogeneity on the trajectory of flower‐size evolution. Allocation theory predicts that in a self‐fertilising plant such as *Arabidopsis thaliana* , petal size should converge towards smaller flowers, reducing both its mean and variance. In contrast, if genetic drift plays a more prominent role (Buysse et al. 2025) or if spatially variable environments maintain divergent selection (MacTavish and Anderson 2020), petal size may remain variable within the species. Leveraging extensive genomic and phenotypic resources, we examine the genetic and ecological drivers of floral‐size evolution following the emergence of selfing and assess how environmental gradients shape these evolutionary trajectories across the species' range. Our results reveal that patterns of phenotypic evolution differ geographically, reflecting variation in selection pressures across environments.

## 2 Materials and Methods

### 2.1 Biological Materials

A subset of 407 *Arabidopsis thaliana* accessions was selected, overlapping the complete range of genetic structure and geographic distribution represented in the 1001 genome project accessions (Alonso‐Blanco et al. 2016) (Figure S1 and Table S1). All accessions were vernalised for 4 weeks in the dark at 5°C in October 2022. Seedlings were then transferred to soil in individual pots, with one plant grown per accession and grown in a glasshouse under semi‐controlled conditions in Montpellier, CEFE CNRS, France (maintained at around 20°C during the day and 15°C at night with natural light). Accessions were randomly distributed on three tables and daily rotated to homogenise the effect of spatial heterogeneity within the glasshouse across the genotypes. Plants were surveyed daily, and the date of first flower opening, flower collection and plant death were recorded. The fourth rosette leaf was collected at bolting and immediately scanned. Three flowers were collected between the 10th and the 25th flower of the main stem from the primary inflorescence. Flowers were collected and preserved in 70% ethanol in individual Eppendorf tubes placed in the dark at room temperature before measurements.

### 2.2 Morphological Measurements

To disentangle petal‐specific growth factors from whole‐flower and general growth factors affecting flower size, we quantified the size of petals, sepals, stamens, seed set and leaves across all accessions. By measuring these additional floral organs and vegetative traits, we aim to assess whether flower size evolves independently of other reproductive and vegetative traits and to control for shared growth trends when isolating the genetic determinants of petal size. Flowers were stained with 0.02% Toluidine Blue for 10 min before being dissected and placed between microscope slides and cover slips with a drop of glycerol (40%, v/v). Slides were scanned at a 4800‐dpi resolution with an Epson perfection v850 pro scanner. Leaf, petal, sepal and short and long stamen sizes (length, width and area when relevant) were measured using Fiji version 2.35 (Schindelin et al. 2012). The blue channel of the images was isolated and contrast‐enhanced with Otsu's local contrast method. Leaves were scanned at 800‐dpi resolution and made binary. Pixels that belong to the organs were counted to estimate organ sizes. Measures were calibrated using the dots per inch (dpi) of the images. The ovule number per flower was counted manually using fluorescence microscopy (Leica DMI4000). Flowering time was calculated as the duration in days from seed sowing to the opening of the first flower. We computed adjusted trait values by extracting the residuals from a linear model that included the relevant confounding factors (petal rank along the stem, stem number and a variable describing spatial distribution in the tables). These adjusted values were used for all downstream statistical analyses, including GWAS. All traits were normally distributed and did not necessitate transformation (Figure S2).

### 2.3 Data Collection From the Literature

Taking advantage of the extensive literature available about the model species, we confronted our phenotype data to published datasets that shared a significant number of accessions. In particular, we used the 107 phenotypes gathered by Atwell et al. (2010), which includes phenology, microbial resistance, plant morphology and ions content values from a variety of experimental setups and the Aradiv database from Przybylska et al. (2023) that contains plant functional strategies related traits measured in a common garden experiment (Pérez‐Harguindeguy et al. 2013). We also used datasets reporting fitness in terms of seed production, where a majority of our accession set was shared, measured in semi‐controlled conditions (greenhouse) (Exposito‐Alonso et al. 2018) and into the wild (Wilczek et al. 2014), and compared our measurement of flower organ size with other available datasets, obtained from very comparable controlled growing conditions (Li et al. 2020; Wiszniewski et al. 2022). In addition, genome‐wide nucleotide polymorphism data from the sampled accessions were obtained from publicly available variant calling format (VCF) data from the 1001 genomes project webpage (https://1001genomes.org/data/GMI‐MPI/releases/v3.1/) (Alonso‐Blanco et al. 2016).

### 2.4 Statistical Analyses

All statistical analyses were performed with R (version 4.3.2). We performed principal component analyses with the package *FactoMineR*. Testing for isometry versus allometry was achieved with standard major axis regression, which assumes an error component from both tested variables, with the package *smatr* (Warton et al. 2006). Skewness and deviation from the average were statistically tested in R using a skewness test and a Student's *t*‐test, respectively. Triangular relationships were tested by comparing statistically the slopes of 5% and 95% quantile regressions (Peng et al. 2017) using the R package *quantreg*.

### 2.5 Genome‐Wide Association Studies (GWAS)

We tested associations between organ size traits and genome‐wide nucleotide polymorphisms using the GEMMA software v0.98.1 (Zhou and Stephens 2012). Only single‐nucleotide polymorphisms (SNPs) were retained in the association analyses, short indels and non‐ACTGN were filtered out, as well as SNPs with a minor allele frequency below 5% using PLINK (Purcell et al. 2007). A total of 1,899,962 SNPs passed the filters. A threshold of 20% maximum missing call was applied, but no genotype was excluded. GEMMA takes the population structure into account by computing and including a relatedness matrix as a covariate in the models. The *p*‐values distribution of all GWAS followed a uniform distribution and did not necessitate transformation for false discovery rate, and genomic inflation factor tended to 1 ± 0.1.

The GEMMA *bslmm* function (Bayesian sparse linear mixed model) was used to estimate the proportion of phenotypic variance explained by the genotypes (PVE, the SNP‐based heritability of the traits) and the Single Nucleotide Polymorphisms effect sizes, as recommended by Zhou et al. (2013). GEMMA fits the equation *Y* = *μ* + *Xβ* + *Xα* + *ε*, where *Y* is the vector of phenotype, *μ* is the intercept (phenotype mean), *X* is the matrix of n individuals times *p* genetic markers, *α* is the vector of small effect that all SNPs have while *β* captures the effect of large effect SNPs. Finally, it estimates *γ*, which is the posterior inclusion probability estimate of *β*, such that the total effect size for a given SNP is *α* + *βγ*.

The GEMMA *lmm* function (linear mixed model) was used to identify specific quantitative trait loci (QTL) associated with the traits. The function tests the null hypothesis *β* = 0 and returns a *p‐value* for each SNP. Despite strong PVE detected notably for petal and sepal size (PVE > 0.7; Table S2), associations rarely exceeded the arbitrary Bonferroni significance threshold accounting for multiple testing. Under the assumption that this is indicative of the interplay of multiple small‐effect QTLs, the threshold of significance was relaxed to consider the potential polygenic architecture of the studied traits. Reports of *p‐values* detecting relevant genes range from 10^–7^ to 10^–4^ (Burton et al. 2007; Chen et al. 2021), but there is no consensus on a threshold to detect multiple minor effect variants in a polygenic architecture (also known as multiple disease phenotype). Because the Bonferroni correction can be overly conservative for traits with a polygenic architecture, we adopted an empirical threshold based on the recovery of the well‐established flowering‐time regulator *FLOWERING LOCUS C* (*FLC*) in the flowering‐time GWAS. The threshold corresponding to this association (*p* = 5.2 × 10^−5^) was subsequently applied to identify candidate loci for other traits. FLC, known as a key regulator of flowering time, is commonly detected in GWAS in *A. thaliana* and its variation accounts for more than half the trait's variation (Sasaki et al. 2018). To eliminate genetically linked variants, we filtered out significant SNPs by considering their genomic position (https://www.arabidopsis.org), statistical significance and predicted function using SnpEff (Cingolani 2022). This process resulted in retaining one focal polymorphism per QTL for subsequent analyses (Table S3).

### 2.6 Population Genetics Analysis

To assess whether the genetic architecture of flower size matches our predictions of directional selection versus drift, we investigated the regime of selection acting on leaf and flower size QTLs across the species distribution with two metrics. We first computed the *πN*/*πS* ratio for all *A. thaliana* annotated genes. We calculated the ratio between the genes' nucleotide diversity in non‐synonymous sites (*πN*—defined as the zerofold degenerate sites) and the nucleotide diversity in synonymous sites (*πS*—defined as fourfold degenerate sites). The rationale is that *πS* gives a measure of neutral genetic variation and serves as a reference for *πN*, which measures the amount of genetic variation at sites under natural selection control. Thus, a value of *πN*/*πS* below 1 is indicative of purifying selection, and a value above 1 is indicative of diverging selection. We used the genome‐wide distribution as a reference: individual genes' *πN*/*πS* included in the 0.05–0.95 quantile do not deviate from the general purifying trend. Student‐test and skew‐test were performed with R to evaluate a set of genes *πN*/*πS* deviation from the average and normality. Full sequences of gene coding regions and general feature format files were downloaded from Phytozome 13 (version Athaliana_447_TAIR10). Together with the VCF file, they were used to generate the diversity of gene sequences observed among *A. thaliana* genotypes. The R package *RGenetics* was used to discriminate fourfold from zerofold degenerate sites, and the *nuc.div* function from *pegas* was used to compute the nucleotide diversity. More details about the methods and scripts used are available at (https://github.com/kevinfrsartori/Whole_Genome_PiNPiS). Second, the extended haplotype homozygosity (EHH) was computed genome‐wide to test whether a particular derived variant is subjected to ongoing or partial sweep in populations (Sabeti et al. 2002). When advantageous mutations are selected, the frequency of genetically linked variants also increases, extending the haplotype length around the selected site. This signal perdures until recombination events erase it. EHH measure the decay of a haplotype length upstream and downstream of the focal SNP, which thus depends on the selection's history and strength. Since the patterns of EHH might also be affected by the species' population structure and demographic history (Voight et al. 2006), we compute this estimate genome‐wide to assess significance of individual loci. The R package *rehh* (Gautier and Vitalis 2012) was used to compute the EHH's test statistic (iHS), defined as the log ratio of the integral of the EHH decay (iHH) around the derived allele divided by the iHH around the ancestral allele. This statistic was then compared to the distribution of iHS for all SNPs of similar allele frequency, and a *p*‐value was reported. The iHS computation relies on the polarisation of the SNP dataset, i.e., the ancestral state of each SNP. To differentiate between derived and ancestral alleles, we used a maximum likelihood method using the focal species allele frequencies together with related species allelic information. We first identified an ortholog gene in *Arabidopsis lyrata* and *Arabidopsis halleri* for most *Arabidopsis thaliana* genes by using OrthoFinder (Emms and Kelly 2015). We kept all *A. thaliana* genes having at least one ortholog and kept only one ortholog per outgroup species based on the genes' distance tree, resulting in a list of 25,411 genes. We extracted a sequence covering the coding region plus the *cis*‐ and *trans*‐regulatory regions for all three gene pairs, and performed a multiple alignment using MAFFT (Katoh et al. 2002). We extracted the allelic information of the outgroup species with custom R scripts and ran the programme est‐sfs (Keightley and Jackson 2018). It is important to note that the computation of iHS was limited to the presence of ortholog genes, resulting in filtering out a few SNPs per trait lists (Table S3).

### 2.7 Habitat Suitability

The *A. thaliana* niche was modelled using the MaxEnt software (Elith et al. 2011; Phillips et al. 2006) by reproducing the method from (Yim et al. 2024) (Figure S3). MaxEnt is a correlative species distribution model that predicts the suitability of an environment for a species based on presence‐only data and a set of environmental variables. Climatic data were downloaded from CHELSA v2.1 (https://chelsa‐climate.org) at a resolution of 30 arc sec (Karger et al. 2017). The species distribution modelling was performed with the following variable panel: the isothermality (ratio of diurnal variation to annual variation in temperatures, °C), the mean of daily minimum air temperature of the coldest month (°C), the annual range of air temperature (°C), mean daily mean air temperatures of the wettest quarter (°C), mean daily mean air temperatures of the warmest quarter (°C), precipitation seasonality (standard deviation of the monthly precipitation estimates, kg m^−2^), mean monthly precipitation amount of the wettest quarter (kg m^−2^ month^−1^) and mean monthly precipitation amount of the driest quarter (kg m^−2^ month^−1^). Altitude data at a resolution of 30 arc sec come from the Global Multi‐resolution Terrain Elevation Data 2010 (GMTED2010) (Danielson and Gesch 2011) and were downloaded from the USGS website. We used the extensive species occurrence data (*N* = 672) provided by Yim et al. (2024), which covers the distribution of the species in an evenly spaced manner (one sample per km^2^) to account for sampling bias. From the MaxEnt model, we estimated the habitat suitability (HS) and the limiting factor (LF) of all *A. thaliana* accessions' collecting sites (Figures S3 and S4). The HS metric scales from zero (unsuitable) to one (favourite combination of environmental clues) for each grid cell, where a higher value indicates environmental condition where *A. thaliana* is likely to occur. LF refers to the climatic variable that has the most significant impact on the prediction of HS when it varies while all other variables are maintained constant (Figure S4). We thus refer to ‘suitable’ or ‘favourable’ habitats as locations with high HS values according to the MaxEnt model based on bioclimatic predictors, and we use the HS value as a general proxy for abiotic resource availability, optimal growing conditions and associated biotic interactions. For each accession, we extracted the HS value at its collection coordinates and used this score as an estimate of how closely its local habitat matches the modelled optimal conditions. The relationships between traits and HS were tested using linear regressions. Linear regressions were plotted when significant, and quantile regressions were plotted when the test for slope difference (Anova) was significant.

### 2.8 Selection Regime Along Habitat Suitability Ranges

In order to test for selection regime shift along the habitat suitability gradient, we divided the *A. thaliana* accession set into 10 HS classes. To avoid the influence of over‐sampling in some regions of the species distribution, we first pruned the accession list based on genetic relatedness. We used the PLINK software to estimate the number of accessions passing a filtering threshold of genetic relatedness ranging from *r* = 0.1 to *r* → 1. We found that the number of related accessions stops dropping after *r* = 0.95 and remains constant while *r* → 1 (Figure S5). We thus excluded 87 accessions with a threshold of *r* = 0.95. To standardise sample size across analyses, HS ranges were obtained by dividing the HS distribution of unrelated accessions into 10 equally sized quantiles, where each quantile contains about 100 accessions. Because the 10 HS classes obtained by splitting *A. thaliana* accessions were defined by habitat suitability rather than geographical or historical population structure, they were not treated as biological populations for classical population‐genetic analyses. Thus, we tested for changes in selection pressure by relying on rare allele frequency only. We used the sequencing dataset presented above and relaxed the minimum allele frequency threshold to a minimum allele count of 1. Only the SNPs having ancestry information located in the gene body were kept, and we computed the unfolded site frequency spectrum (uSFS) for leaf and flower organs associated genes. The allele counts for ranges of allele frequency of 0.05 were reported. Since new mutations are putatively deleterious, natural selection maintains new allele frequencies at a low level. As a result, uSFS are right‐skewed distributions of allele counts, with a large number of low‐frequency mutations. A reduction in uSFS skewness is therefore consistent with reduced intensity or efficacy of purifying selection.

## 3 Results

### 3.1 Unsuspected Variation of Flower Size in *A. thaliana* Is Highly Heritable

**FIGURE 1:**
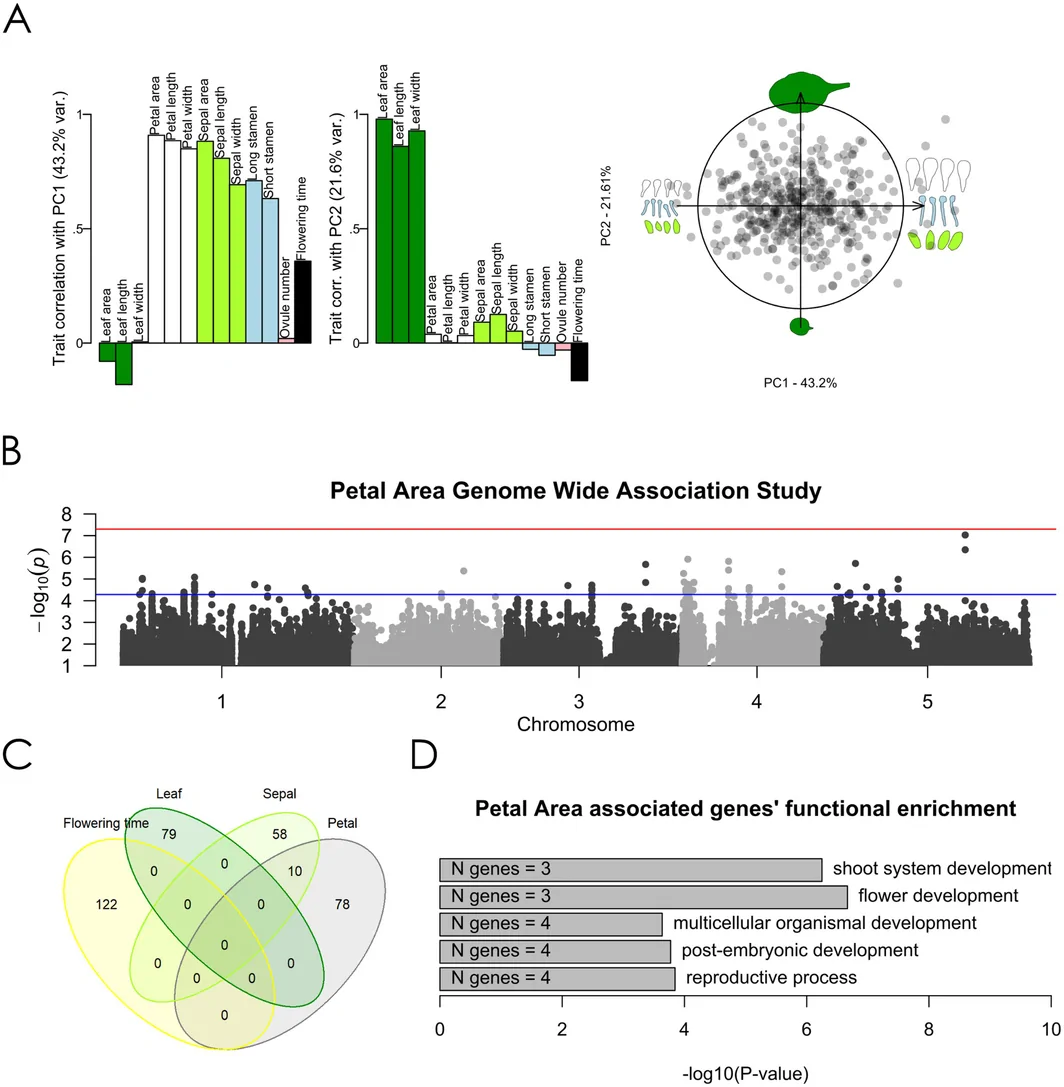
Extensive flower size variation in *A. thaliana* is determined by a polygenic architecture of low pleiotropic mutations. (A) A Principal component analysis (PCA) was performed on flower organs and leaf size excluding flowering time that was correlated post hoc and represented as a supplementary variable. Bar plots show trait correlations with principal components (PC), with colours indicating different organs: Dark green, leaf; light green, sepal; white, petal; blue, stamen; pink, ovules. The biplot displays accession coordinates along the first two principal components (PC), with organ silhouettes representing extreme organ size values at the same scale. (B) Manhattan plot of the petal area genome‐wide association study. Red line represents the Bonferroni threshold, and the blue line represents the relaxed polygenic threshold. (C) Venn diagram illustrating the lack of overlap between genes associated with leaf and flower organs' size and flowering time variation. (D) Representation of the gene ontology functional enrichment of petal area associated genes. Only significant terms are represented, including gene number and *p*‐value of a Fisher exact test.

The length, width and area of floral organs and leaves showed substantial variation across 407 *A. thaliana* accessions spanning the species' global genetic and geographic diversity (Figures S1 and S2 and Table S1). Floral organ size explained most phenotypic variance among accessions (43.2%, PC1; Figure 1A), and all floral traits were strongly correlated with the first principal component (*r* > 0.5). Leaf size variation was captured primarily by PC2 (21.6% of the variance), while ovule number contributed mainly to PC4 (< 8% of variance). Petal area showed the greatest variability, matching leaf area in its coefficient of variation and spanning more than a threefold range (Table S2).

We next assessed how much of the observed flower‐size variation in our experiment reflects genetic differences by computing trait heritability and correlating our measures with independent, publicly available flower‐diameter estimates. Variance‐based heritability estimates (Table S2), accounting for trait variance both within and between regional populations of *Arabidopsis thaliana* (Alonso‐Blanco et al. 2016), revealed high heritability for all investigated traits. In particular, petal and sepal traits showed very high values (*H* ^2^ > 0.8). Moreover, our measurements of flower organ sizes were significantly correlated with measures of flower diameter across temperature treatments (Spearman's rank correlation coefficient≈0.46), indicating that relative differences among genotypes are largely preserved across environments (Wiszniewski et al. 2022). Together, these results indicate that genetic variation accounts for a significant part of flower organ size variance across *A. thaliana* worldwide genotypes.

These results indicate that floral‐size variation in *A. thaliana* remains substantial despite selfing, consistent with either incomplete fixation of size‐reducing alleles during the evolution of selfing or environmentally structured selection maintaining divergence among lineages. Combined with the high heritability estimates, this suggests that the observed phenotypic differences largely reflect stable genetic differences among accessions, supporting the use of this dataset to dissect the genetic basis of natural variation in petal size.

### 3.2 Independent, Low‐Pleiotropy Mutations Largely Account for the Variation in Flower Size

We investigated whether indirect effects, such as linked selection (Smith and Haigh 1974) or pleiotropy with other traits under selection, drive the evolution of floral size in *Arabidopsis thaliana* . To this end, we first tested for phenotypic covariation between floral organ size and other traits among genotypes (Table S2). Stamen, petal and sepal size were significantly and positively correlated (*r* = 0.28–0.93) but showed no correlation with ovule number or leaf size, except for a weak negative correlation between leaf length and petal width (*r* = −0.15; Figure S6). The relationship between long‐stamen length and petal length was strictly isometric (slope = 1, *p* > 0.01), suggesting an overlap in their genetic bases, while other pairwise relationships were allometric (slope < 1, *p* < 0.001). All floral organs were positively correlated with flowering time, although the correlation coefficient did not exceed 0.5 (*r* = 0.14–0.42). We further examined correlations with published phenotypes. Floral organ size traits showed no significant correlation with any of the 107 phenotypes from Atwell et al. (2010) (Table S2). Specific leaf area (SLA), leaf nitrogen content (LNC) and total plant biomass (Przybylska et al. 2023) were significantly correlated with several floral size traits from our dataset (*r* = −0.35 to 0.4). Most traits from Przybylska et al. (2023) were also correlated with our measurement of flowering time (*r* = −0.67 to 0.70). Although these datasets were not generated under identical environmental conditions, the lack of strong cross‐dataset correlations is consistent with at most modest pleiotropic effects of mutations controlling flower size, especially given the high heritability and large genetic contribution of floral organ size.

To test more formally for shared genetic bases, we quantified genetic correlations using multivariate GWAS (mGWAS) for significantly correlated trait pairs. The mGWAS partitions genetic variance into trait‐specific and shared components, allowing estimation of the genetic correlation (*r*_g_) between traits (Zhou and Stephens 2012) (Table S4). Within our dataset and published datasets (Atwell et al. 2010; Przybylska et al. 2023), we detected no significant genetic correlations between floral and most leaf traits, indicating independent genetic bases. Petal and sepal areas showed, nevertheless, weak but significant correlations with LNC and plant dry mass (*r*_g_~0.3, *p* < 0.01), which are proxies for resource acquisition ability (Pérez‐Harguindeguy et al. 2013). These results suggest that resource acquisition capacity modestly influences both vegetative carbon economy and floral organ growth, supporting a role for allocation constraints in flower‐size evolution. Overall, floral organ size variation showed limited phenotypic and genetic correlation with other traits, indicating that pleiotropy and linked selection exert only minor influence on floral size evolution in *A. thaliana* .

We next conducted univariate GWAS to identify loci underlying individual trait variation (Figure 1B). We identified 61, 105, 87, 59, 38 and 65 SNPs, corresponding to 144, 191, 194, 126, 117 and 122 candidate genes, associated with leaf, petal, sepal, stamen size, ovule number and flowering time, respectively (Figure 1C and Table S3). Functional annotation using TAIR gene ontology terms (TAIR, Huala et al. 2001; Berardini et al. 2004) identified several known growth regulators among candidate genes, including *JAGGED*, *GROWTH‐REGULATING FACTOR 6*, *CYTOCHROME P450 90B1*, *KINASE‐INDUCIBLE DOMAIN INTERACTING 9*, *E2FE*, *MYB DOMAIN PROTEIN 104*, *RESPONSE REGULATOR* 7, *INDOLE‐3‐ACETIC ACID INDUCIBLE 19* and *SEEDSTICK* (Dubos et al. 2010; Gómez‐Ocampo et al. 2024; Gonzalez et al. 2015; Lee et al. 2008; Lopes et al. 2023; Maki et al. 2022; Omidbakhshfard et al. 2015; Sauret‐Güeto et al. 2013; Vlieghe et al. 2005). Genes associated with petal area were significantly enriched for functions related to floral and shoot development (six genes, *p*~0.01) and reproductive regulation (12 genes, *p*~0.02; Figure 1D), thereby supporting our ability to detect the polygenic architecture of the trait (Table S5). We observed no overlap between SNPs and genes associated with floral and leaf area, consistent with the absence of phenotypic and genetic correlations (Figure 1C). However, we detected among petal area‐associated genes, the MADS‐box transcription factor FLOWERING LOCUS M (FLM), first identified as a regulator of flowering and later associated with more complex pleiotropic effects on plant organ growth (Hanemian et al. 2020). Despite moderate phenotypic correlations among floral organs, their genetic determinants showed limited overlap. The observed phenotypic covariation is likely reflecting coordinated responses to shared environmental conditions rather than shared genetic architecture. Thus, floral organ size appears to evolve largely independently via low‐pleiotropic mutations.

### 3.3 Flower‐Size Genes Are Under a Mixed Selection Pattern in *Arabidopsis thaliana*

**FIGURE 2:**
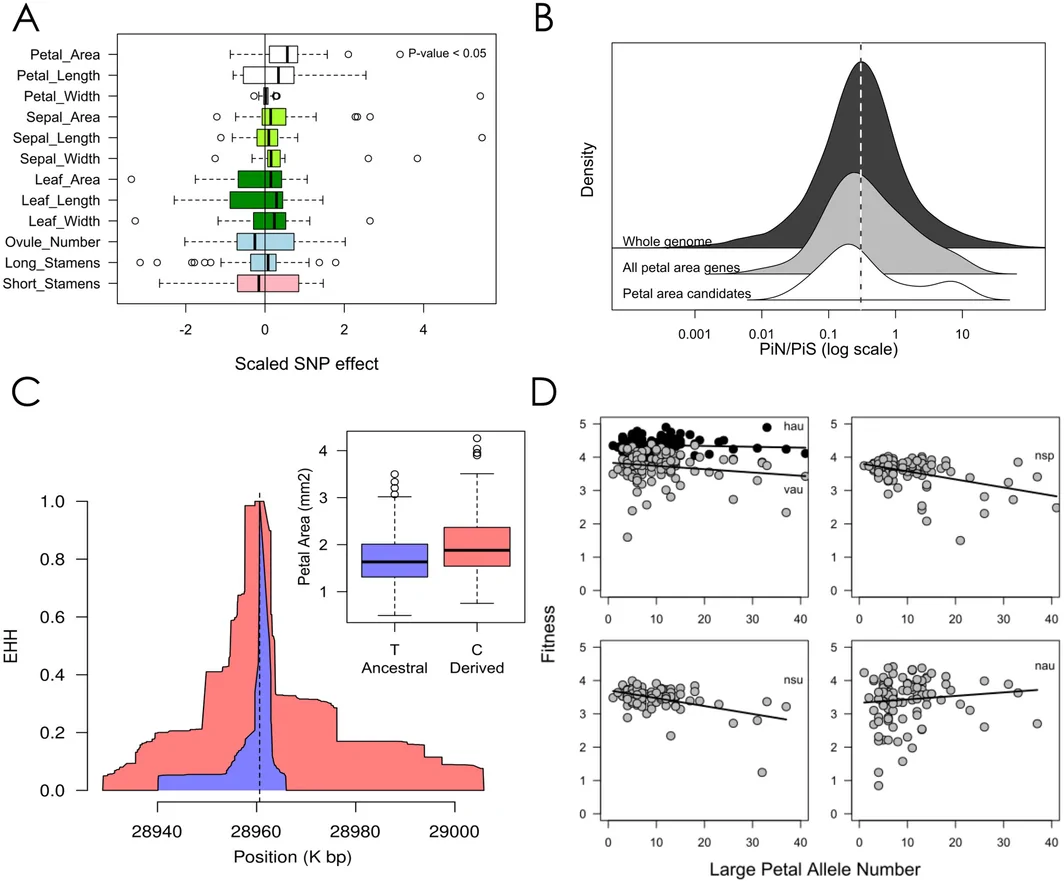
A habitat‐dependent flower size–seed production trade‐off explains a mixed selection pattern on flower size in *A. thaliana* . (A) Distribution of derived alleles' total effect size from each associated SNPs identified in GWAS for each trait investigated. A *p*‐value is given for trait distributions significantly different from 0 (vertical line). (B) *πN*/*πS* distribution across all *Arabidopsis thaliana* genes, among the petal area genes and a priori candidate genes identified in the GWAS for petal area. The dashed line represents the whole genome average. (C) Extended haplotype homozygosity around FLM lead SNP position. The red and blue integrals represent the degree of haplotype identity shared by individuals carrying the C (derived) and T (ancestral) alleles, respectively, as a function of distance to the lead SNP (dashed line). (D) Relationship between the number of large petal alleles and seed production across different experimental fields. hau, Halle autumn; nau, Norwich autumn; nsp, Norwich spring; nsu, Norwich summer; vau, Valencia autumn. Black lines represent significant fits (ANOVA) of linear regressions.

Contrary to the expectation that resources should be reallocated away from floral investment in selfers, more than three‐quarters of the derived alleles associated with petal area variation had a positive effect on size (Figure 2A). Moreover, this excess of positive effects among derived alleles was unique to petal area. We further investigated whether it is a consequence of neutral processes or whether these alleles are under selection.

To assess whether the fixation of derived alleles increasing flower size could result from genetic drift, we first computed genome‐wide *πN*/*πS*, which contrasts the accumulation of non‐synonymous (*πN*) and synonymous (*πS*) diversity to infer the strength and direction of selection. Genes containing SNPs associated with petal area exhibited a skewed *πN*/*πS* distribution that tended to fall below the genome‐wide average (*p* < 0.1), indicating ongoing purifying selection rather than relaxed selection (Figure 2B). Other floral organ size traits showed no comparable pattern (Figure S7). Nonetheless, the *πN*/*πS* distribution of petal area genes was bimodal, with most genes shifted towards lower *πN*/*πS* compared to the genome‐wide average, while three genes, *AT1G36940*, *KIX9* and *FLM*, show elevated values within the top 5% of the genome‐wide distribution. This pattern is consistent with diversifying selection acting on the protein sequences of only a few of the petal size candidates.

We next examined whether derived alleles in petal area genes show evidence of positive selection. At a bi‐allelic site, alleles under selection are expected to be associated with longer haplotypes due to incomplete selective sweeps. We estimated haplotype decay around each focal SNP using genome‐wide extended haplotype homozygosity (EHH) (Klassmann and Gautier 2022). The standardised ratio of derived to ancestral haplotype homozygosity (the integrated haplotype homozygosity score, iHS) identifies alleles with unusually long haplotypes for their frequency. Two petal area SNPs exhibited high |iHS| values, resulting from an extended haplotype homozygosity around the derived alleles, a sign of recent positive selection. The first, snp_1_10937139 (*p* = 0.013), increased petal area and likely affects the growth regulator JMJ18. The second, snp_5_870273 (*p* = 0.02), decreased petal area and likely influences AT5G03480, a poorly characterised RNA‐binding protein expressed during early petal development (Klepikova et al. 2016). A third SNP, snp_1_28960616 (*p* = 0.07), showed a marginal signal of positive selection and likely affects FLM, a known flowering‐time regulator that interacts with SVP to influence floral morphology (Figure 2C) (Posé et al. 2013). As a control, we repeated the analysis for all floral and leaf traits and quantified the enrichment of trait‐associated SNPs within the top 0.05% of the genome‐wide iHS distribution (Tsuchimatsu et al. 2020). Leaf size‐associated SNPs showed a threefold enrichment, whereas no enrichment was detected for flower size (Figure S8).

Together, these results indicate a lack of consistent directional selection acting on the genetic determinants of flower size in *A. thaliana* . Neither purifying nor positive selection acts uniformly across petal size regulators, and no consistent directional trend is observable.

### 3.4 The Influence of Flower Size on Individual's Performance Is Environment Dependent

To understand the lack of general unidirectional selection pattern, we investigated the effect of environmental heterogeneity by testing the relationship between petal size variation and plant fitness across populations and environments. We used fitness data from common garden experiments conducted at three sites, Valencia (Spain), Norwich (United Kingdom) and Halle (Germany) (Wilczek et al. 2014). Among the candidate SNPs associated with petal area, we counted the number of positive effect size alleles carried by each genotype, which provides a proxy for the genetic predisposition to produce larger petals. Consistent with expectations of the selfing syndrome, genotypes carrying more large‐petal alleles generally performed worse than those carrying fewer large‐petal alleles across most environments (Figures 2D and S6).

However, the relationship between the number of large petal alleles and fitness varied across sites and seasons. The relationship was negative in spring and summer in Norwich, but weakened in autumn in Halle and Valencia, and shifted to a positive relationship in autumn in Norwich. Under more controlled experimental conditions, the negative relationship was significant in Madrid and Tuebingen in control treatments, whereas it weakened and became nonsignificant under drought treatments at both sites (Figure S9).

Overall, these results suggest that individual performance is affected by the genetic propensity to invest in petal growth, but the strength and form of this relationship depend on environmental conditions. Such context dependence likely generates heterogeneous selection on petal size across the geographic range of *A. thaliana* , possibly explaining the lack of unidirectional selection at the species level.

### 3.5 Habitat Suitability Influences the Selection Regime at Flower Size Loci

To examine further the environmental constraints on flower size evolution, we quantified habitat suitability at the collection sites of the studied populations. The top 5% of habitat suitability sites correspond to narrower ranges of bioclimatic variables and altitude (hereinafter referred to as niche climatic centre), compared to the 5% tail of habitat suitability sites, which were associated with more extreme values, above and below (niche climatic edges) (Table S6). First, we examined how the mean petal area and its variance change along a climatic habitat‐suitability (HS) gradient. Second, we exploited the knowledge of the genetic basis of petal‐size variation to infer spatial variation in selection acting on flower‐size loci independently of direct floral measurements.

**FIGURE 3:**
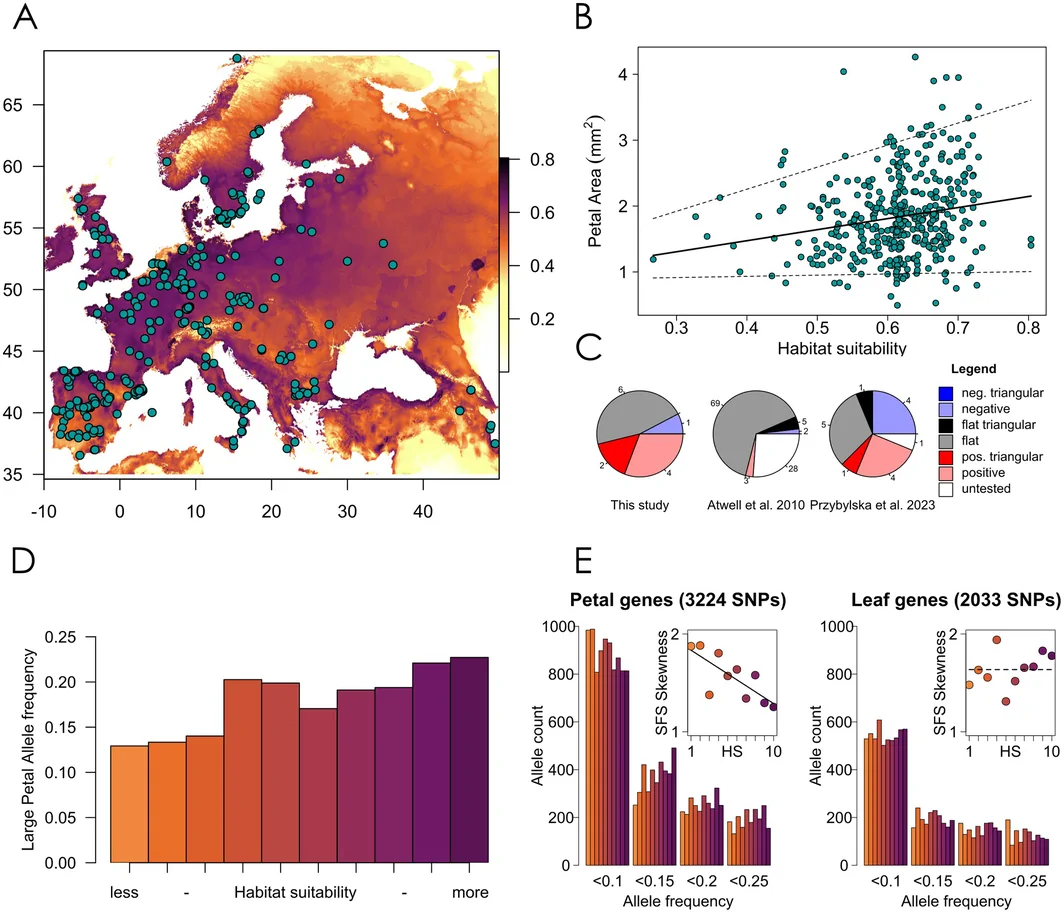
Habitat suitability shapes patterns of phenotype diversification across the geographical range of *A. thaliana* . (A) Map of the studied European accessions. The orange to purple scale represents the modelled habitat suitability from less to more suitable, and the blue dots represent accessions' collecting sites. (B) Correlation between petal area and habitat suitability. Lines represent significant regressions: Plain line, linear model; dashed lines, 0.05 and 0.95 quantile regressions. (C) Relative proportion of different types of significant relationships between traits and habitat suitability in three independent datasets. (D) Large petal alleles' frequency along habitat suitability clusters. (E) Unfolded site frequency spectrum (SFS) among SNPs located in petal and leaf gene sets. The colours of the bars correspond to the habitat suitability classes. Inserts represent the relationship between the SFS's shape and habitat suitability, where a plain line depicts significant and a dashed line insignificant correlation.

Across populations, petal area was positively associated with habitat suitability (Figure 3B). However, the relationship was triangular rather than linear: petal size variance increased towards more suitable environments, mirroring the previously described relationship between flowering time and habitat suitability (Yim et al. 2024). Quantile regression confirmed this pattern, with the 0.05 and 0.95 quantile slopes differing significantly. This indicates that petal size evolution is more constrained at the climatic margins of the species' range, where environmental stress limits expansion (Yim et al. 2024), while selection on flower size is more relaxed in favourable habitats.

To assess whether this triangular pattern is specific to petal area, we tested the relationship between habitat suitability and trait variance in multiple datasets (Atwell et al. 2010; Przybylska et al. 2023) (Figure 3C). None of the 107 traits in Atwell et al. (2010) showed significant heteroscedasticity, and only whole‐plant dry mass in Przybylska et al. (2023) exhibited a similar positive triangular pattern. In our dataset, sepal length, in addition to petal area, displayed both a positive and triangular relationship with habitat suitability. Several other traits showed consistent but homoscedastic responses, including positive correlations for sepal size (this study), flowering time and leaf dry mass (Atwell et al. 2010; Przybylska et al. 2023) and negative correlations for leaf length, rosette diameter, fruit length and leaf nitrogen content. Together, these results suggest that plant growth and life‐history traits broadly align with habitat suitability, and that variance in floral organ size is particularly reduced under less favourable conditions.

To test whether these phenotypic patterns are mirrored at the genetic level, we analysed how alleles associated with petal size, identified by GWAS, are distributed along HS classes. Consistently with the phenotypic trend, large‐petal alleles increased in frequency in more suitable environments (Figure 3D). We tested whether this could reflect variation in purifying selection intensity across the species' range. Purifying selection suppresses deleterious mutations, leading to an excess of rare derived alleles and a skewed site frequency spectrum (SFS). We computed the unfolded SFS for genes associated with petal and leaf traits to compare these effects. Low‐frequency derived alleles (0.05 < frequency < 0.1) represented about one‐fourth of SNPs in both gene sets (Figure 3E). The SFS of petal genes displayed significantly higher skewness in low‐suitability habitats, indicating stronger purifying selection at the environmental margin. By contrast, SFS distributions for leaf genes did not differ along the gradient. Together, the phenotypic (HS‐petal area relationship) and genetic (allele‐frequency shifts at petal‐size loci) trends are cumulative evidence that selection on flower size is stronger and more constraining at the species' climatic margins and more relaxed in favourable habitats, consistent with our independent fitness‐based analyses.

## 4 Discussion

Contrary to predictions from resource allocation theories, our findings indicate that petal size variation in *Arabidopsis thaliana* is not governed by a uniform trade‐off with fitness. Instead, we uncovered a diversity of local selection pressures. Producing large flowers can be costly, particularly in a species capable of generating up to a thousand flowers (Exposito‐Alonso et al. 2018). Yet, this constraint appears most pronounced at the margins of the species' climatic range, where it favours small flowers. In contrast, in more favourable environments, the genetic propensity for larger petal size appears to be partially decoupled from fitness, allowing mutations that increase petal size to persist or even spread. Such spatial variation in selection likely contributes to the greater phenotypic variance in flower size among genotypes from climatically suitable regions.

The lack of genetic correlation and overlap in association signals suggests that petal size has largely evolved independently from other traits. However, our results do not completely exclude a role for life‐history traits, such as flowering time, in petal size variation through pleiotropic effects. We detected a weak phenotypic correlation between flower size and flowering time, a trait known to be under positive selection in *A. thaliana* (Fournier‐Level et al. 2022). Interestingly, among the few genes showing signatures of relaxed purifying selection and selective sweeps was *FLM*, a key regulator of flowering time that also influences plant growth (Hanemian et al. 2020). As *FLM* integrates temperature signals to control flowering, the absence of a clear genetic association with flowering at this locus does not rule out that selection on flowering time may indirectly affect flower size through the pleiotropic effects of shared regulatory pathways. Nevertheless, the highly polygenic nature of petal size, together with the identification of growth‐regulator candidates in genetic association, suggests that pleiotropy is unlikely to be a major determinant of flower size variation.

Although our study does not explicitly partition variance into genotype, environment and genotype‐by‐environment components, previous work on Arabidopsis flower size (Wiszniewski et al. 2022) shows that temperature‐mediated *G* × *E* is clearly present but does not dominate trait variance. Consistent with this, petal size in our experiment is highly heritable within a given environment (*H* ^2^≈0.8), and genotype rankings show moderate but significant consistency across experiments (*R* = 0.35, *p* < 0.01 for 17°C and *R* = 0.46, *p* < 0.01 for 23°C) despite differences in trait definition (flat petal area versus flower diameter) (Wiszniewski et al. 2022). Together, these observations indicate that flower size has a strong and stable genetic component, while genotype‐by‐environment interactions and environmental conditions also contribute appreciably to phenotypic variation. Moreover, the selective signatures observed at genes controlling flower size parallel the phenotypic trends, suggesting that functional constraints on small‐flower alleles intensify towards the species' climatic limits.

In addition to a relaxation of purifying selection, we detected evidence of positive selection acting on derived large‐petal alleles, particularly in environmentally favourable regions. This suggests that an increase in petal size may confer adaptive advantages under certain conditions, or at least when environmental constraints on performance are minimal. The adaptive value of these alleles may relate to the persistence of a low but nonzero rate of cross‐fertilisation in *A. thaliana* (Abbott and Gomes 1989; Hoffmann et al. 2003), potentially favoured during post‐glacial expansions northward, following secondary contact in the Iberian Peninsula (Fulgione and Hancock 2018). Until today, the natural variation of selfing rate (s) across a large sample of *A. thaliana* populations has been reported once (Platt et al. 2010). Consistently, we detected a positive correlation between the outcrossing rate (log(1 − *s*)) and petal area (*R* = 0.25, *p* = 0.12) and petal length (*R* = 0.32, *p* = 0.05) (Figure S10). While suggestive, these relationships should be interpreted cautiously due to the modest sample size (overlapping genotypes, *N* = 39) and the multiple testing context and will require validation through targeted analyses.

Other ecological factors may also promote larger flowers and should be explored in future studies, including historical interactions with floral enemies (Galen 1999; Teixido et al. 2011), protection against microbial and viral infections (Vannette 2020) and the role of flower thermogenesis in pollen germination (van der Kooi et al. 2019). Pleiotropy and linked selection could further contribute to the emergence of large‐flower alleles, especially as some petal size‐associated variants lie within genes that influence other organs or developmental pathways (Hanemian et al. 2020). While our genetic correlation analyses indicate that such effects are limited, functional validation of causal mutations will be necessary to strictly disentangle developmental coupling from direct adaptive responses.

Overall, this study reveals striking geographic variation in investment in reproductive structures, particularly flower size, across the range of *A. thaliana* . Classic theory predicts that selfing lineages experience strong selection for reduced flower size, leading to the fixation of small‐flower alleles due to their lower resource costs. Our results support this expectation only under suboptimal conditions, where environmental constraints and possibly resource limitation likely intensify selection for smaller flowers. In contrast, in favourable environments, relaxed purifying selection reduces these constraints, enabling the persistence or even evolution of larger‐flowered variants. Together, these findings illustrate how environmental heterogeneity modulates the balance between selection and constraint, shaping the trajectory of phenotypic evolution in selfing species.

## Author Contributions

Conception and design: Kevin Sartori, Denis Vile, Francois Vasseur, Cyrille Violle and Adrien Sicard. Data acquisition: Clàudia F. Mestre, Md Jonaid Hossain, Aurélien Estarague, Elza Gaignon, Pierre Moulin and Jesse R. Lasky. Data analysis: Kevin Sartori, Clàudia F. Mestre, Md Jonaid Hossain and Adrien Sicard. Interpretation of data: Kevin Sartori, Clàudia F. Mestre, Md Jonaid Hossain, Aurélien Estarague, Elza Gaignon, Pierre Moulin, Jesse R. Lasky, Denis Vile, Francois Vasseur, Cyrille Violle and Adrien Sicard. Writing: Kevin Sartori, Clàudia F. Mestre, Md Jonaid Hossain, Aurélien Estarague, Elza Gaignon, Pierre Moulin, Jesse R. Lasky, Denis Vile, Francois Vasseur, Cyrille Violle and Adrien Sicard. Funding acquisition: Adrien Sicard and Jesse R. Lasky.

## Funding

This work was supported by the Swedish Research Council (2018‐04214) and the Novo Nordisk Foundation (NNF22OC0079830) to A.S. and J.R.L. was supported by NIH award R35GM138300.

## Ethics Statement

The authors declare that the study was held following Wiley's best practice guidelines on research integrity and publishing ethics. They declare that they did not use artificial intelligence tools or technologies for the realisation of this work. All participants of the study, from data collection to statistical exploration, no matter their degree, were offered participation in the result discussion and article writing and were included as coauthor upon. All authors approve the manuscript and declare no conflict of interest.

## Conflicts of Interest

The authors declare no conflicts of interest.

## Supporting information


**Figure S1:** Geographic distribution and genetic differentiation of *Arabidopsis thaliana* accessions.
**Figure S2:** Distribution of the traits measured for this study.
**Figure S3:** Predicted habitat suitability for *Arabidopsis thaliana* across emerged lands.
**Figure S4:** Geographic distribution of the factors limiting the habitat suitability and their effect on petal area.
**Figure S5:** Genetic clones in the 1135 accessions set.
**Figure S6:** Pairwise relationships between organ sizes.
**Figure S7:** ΠN/πS distribution from genes associated with leaf and flower traits, and flowering time.
**Figure S8:** Fold enrichment of SNPs associated with a trait, among the most significant SNPs on a genome wide scan for selection (Extended Haplotype Homozygosity).
**Figure S9:** Fitness as a function of large petal allele number across *Arabidopsis thaliana* accessions.
**Figure S10:** Outcrossing rate as a function of petal area and petal length.

**Table S1:** Genotype mean traits values.

**Table S2:** Flower and leaf trait variation, and covariation with other studies.

**Table S3:** SNP lists associated with flowering time, flower and leaf traits.

**Table S4:** Genetic correlation matrix.

**Table S5:** Gene ontology (GO) enrichment analyses.

**Table S6:** Ranges of bioclimatic variables associated to *A. thaliana* collection sites.

## Data Availability

All new phenotyping data produced for this study are available in the supplementary files attached to the article. The complete set of scripts used to perform the statistical analyses and the figures is available online at https://github.com/kevinfrsartori/. All sequencing data and genome assembly used in this study were previously published and are available online.
