# Supplementary material for: Local Trade‐Offs Shape Flower Size Evolution Across Arabidopsis thaliana Distribution
Source: Mol Ecol. 2026 Aug 28;35(17):e70525. doi: 10.1111/mec.70525 (PMC13523003; doi:10.1111/mec.70525)
Supplement: Supplementary file 1 [file MEC-35-e70525-s006.pdf]

MOLECULAR ECOLOGY

Supplemental Information for:

Local trade-offs shape flower size evolution across Arabidopsis thaliana distribution.

Kevin Sartori, Clàudia F. Mestre, Md Jonaid Hossain, Aurélien Estarague, Elza Gaignon, Pierre Moulin, Jesse R. Lasky, Denis Vile, Francois Vasseur, Cyrille Violle and Adrien Sicard.

Table of Contents:

**Figure S1**	Page 2
**Figure S2**	Page 3
**Figure S3**	Page 4
**Figure S4**	Page 5
**Figure S5**	Page 6
**Figure S6**	Page 7
**Figure S7**	Page 8
**Figure S8**	Page 9
**Figure S9**	Page 10
**Figure S10**	Page 11
**References**	Page 12

MOLECULAR ECOLOGY

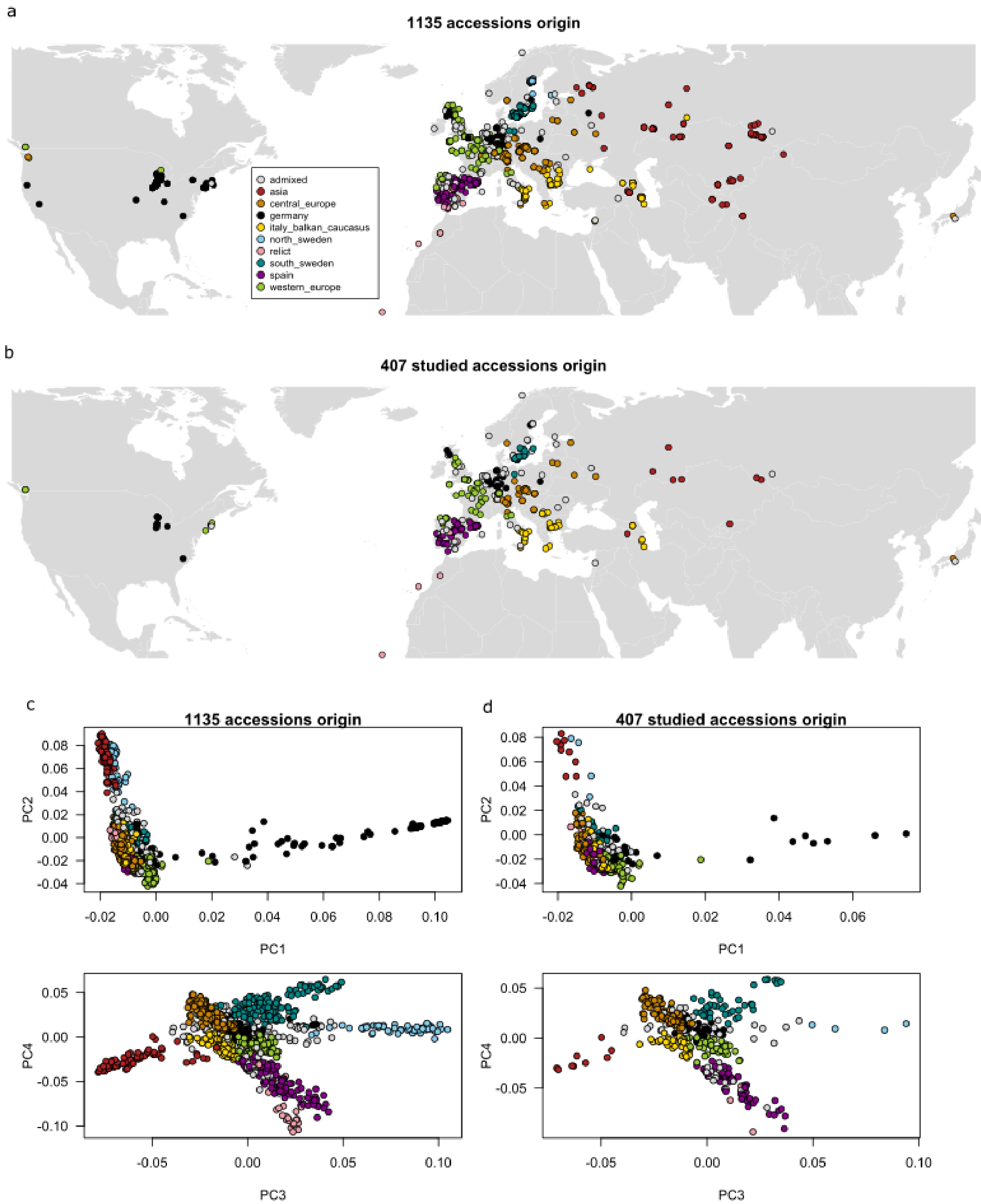

Figure S1. Geographic distribution and genetic differentiation of *Arabidopsis thaliana* accessions. a) Geographic distribution of all sequenced accessions form the 1001 genome project (N=1135). b) Distribution of the herein studied accessions (N=407). c) and d) Distribution of the accessions on the four principal components of their genetic variation. Colors refer to the admixture analysis performed in the original paper from the 1001 genome project (Alonso-Blanco et al., 2016), and are reported on the above map.

MOLECULAR ECOLOGY

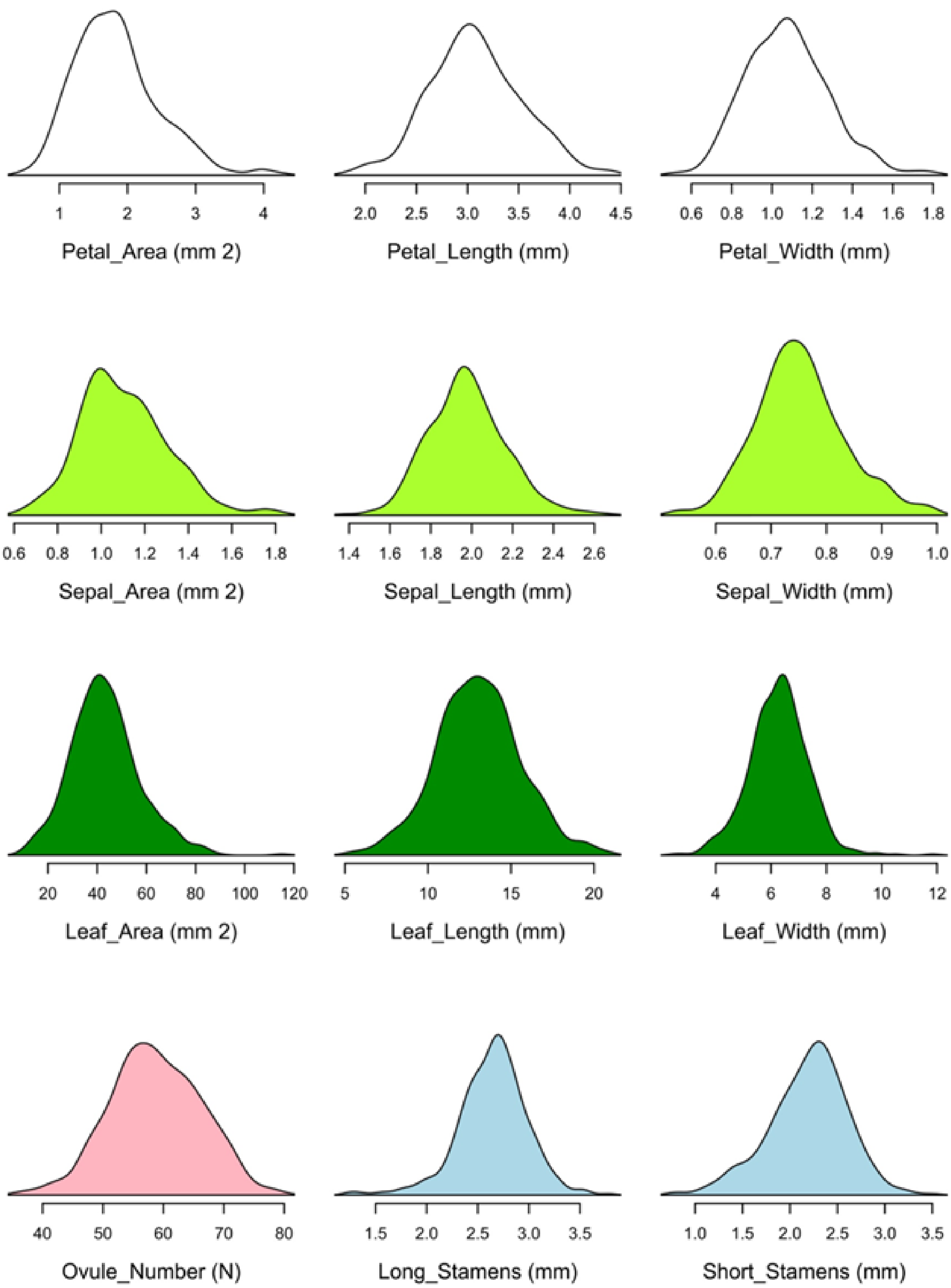

Figure S2. Distribution of the traits measured for this study. Colors represent only the color code used throughout the manuscript.

MOLECULAR ECOLOGY

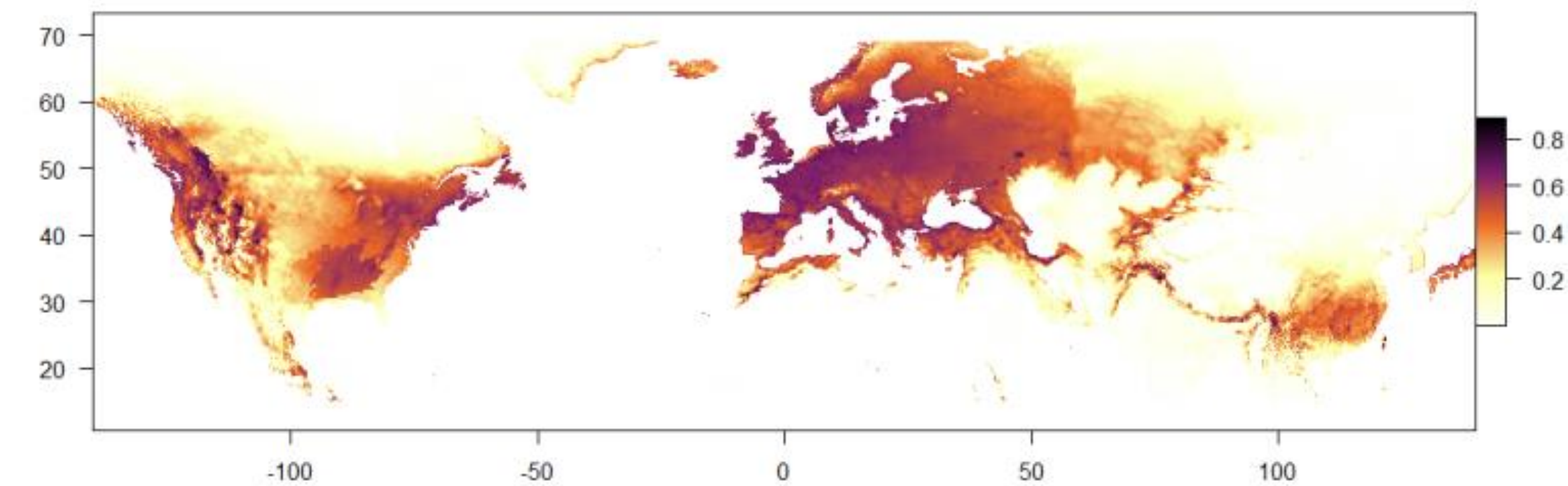

Figure S3. Predicted habitat suitability for *Arabidopsis thaliana* across emerged lands. The color shade from light yellow to dark red represent the habitat suitability metric, that scales respectively from zero (unsuitable) to one (favourite combination of environmental clues) for each grid cell.

MOLECULAR ECOLOGY

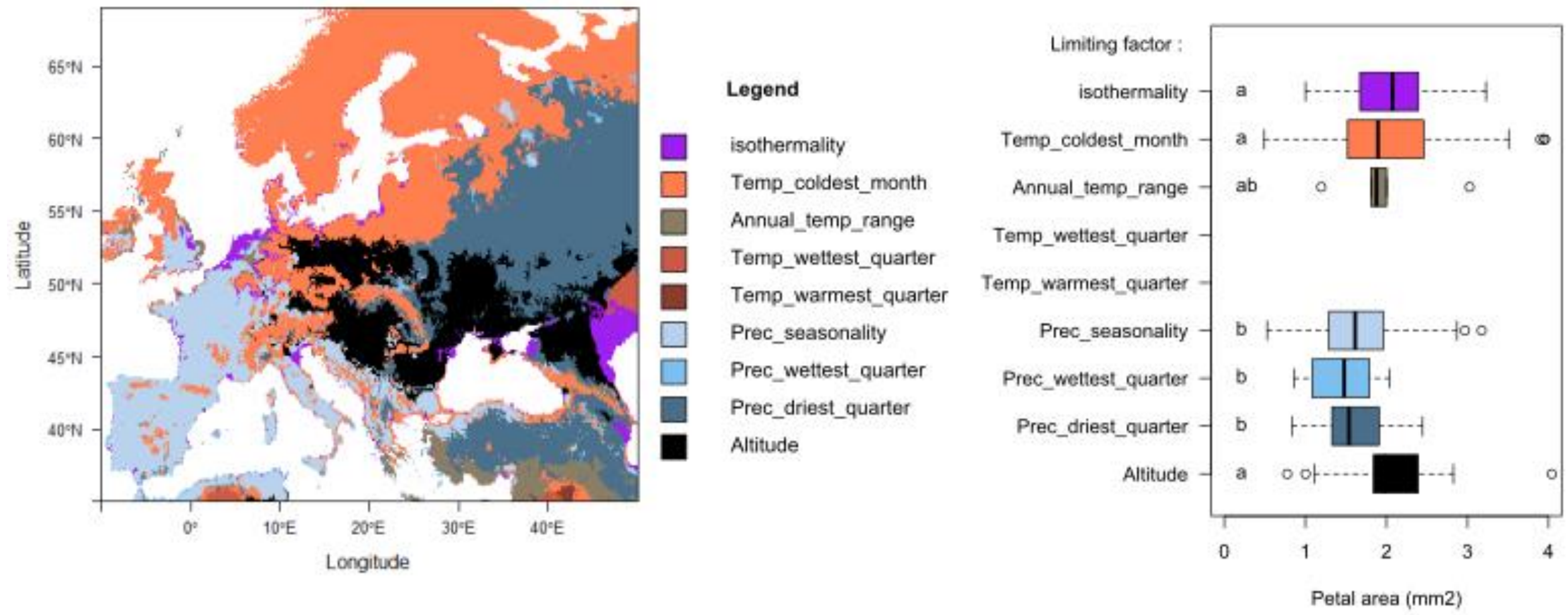

Figure S4. Geographic distribution of the factors limiting the habitat suitability and their effect on petal area. a) Distribution of the limiting factors over Europe; b) Boxplots showing the distribution of petal area among accessions split by the limiting factor of their collecting site. Letters indicate significant differences determined by one-way ANOVA with Tukey's HSD test (α=0.05).

MOLECULAR ECOLOGY

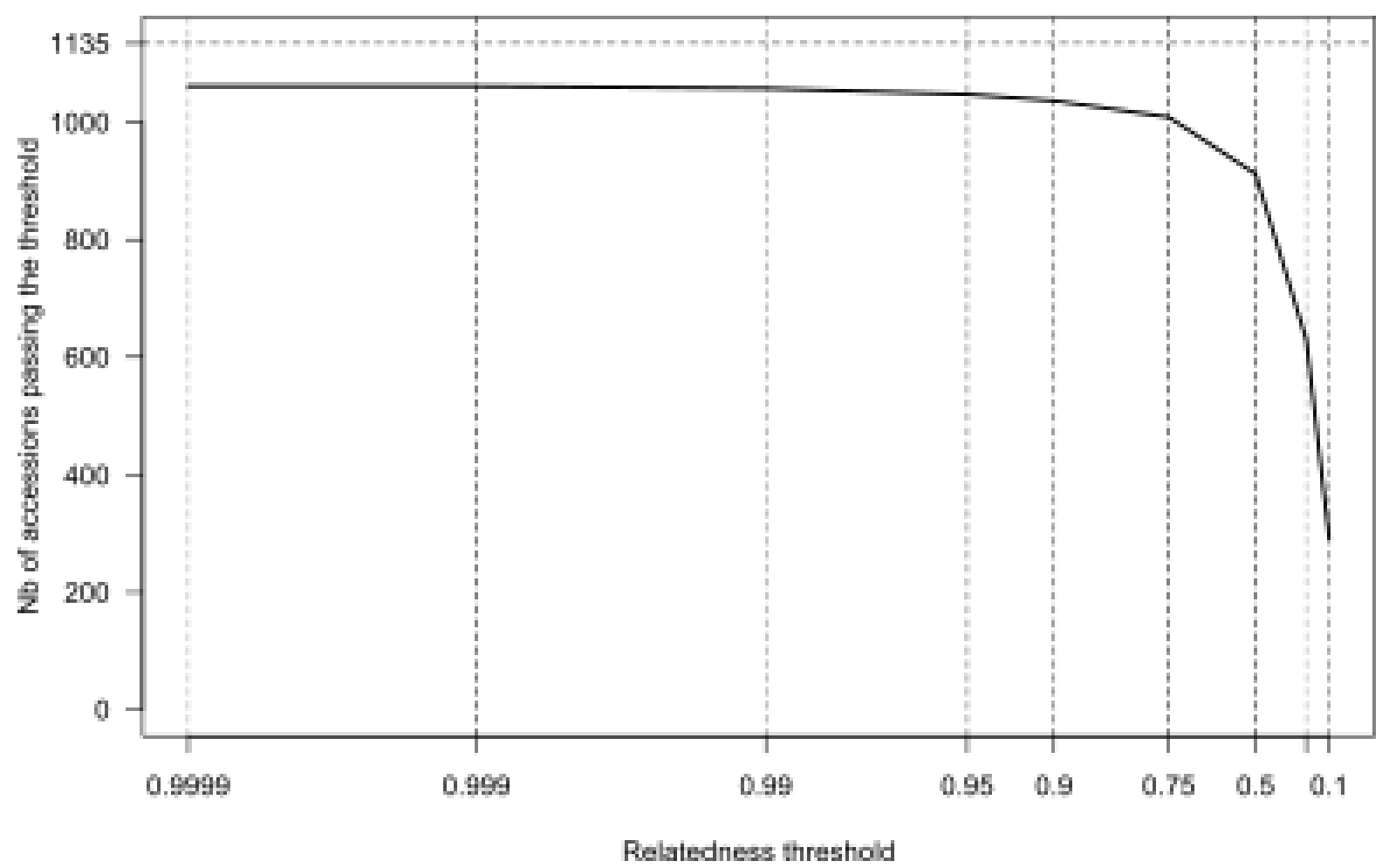

Figure S5. Genetic clones in the 1135 accessions set. A relatedness matrix computed on the SNP dataset was used to filter out genetic clones" based on a threshold (x-axis) and the number of accessions passing the threshold was reported (y-axis).

MOLECULAR ECOLOGY

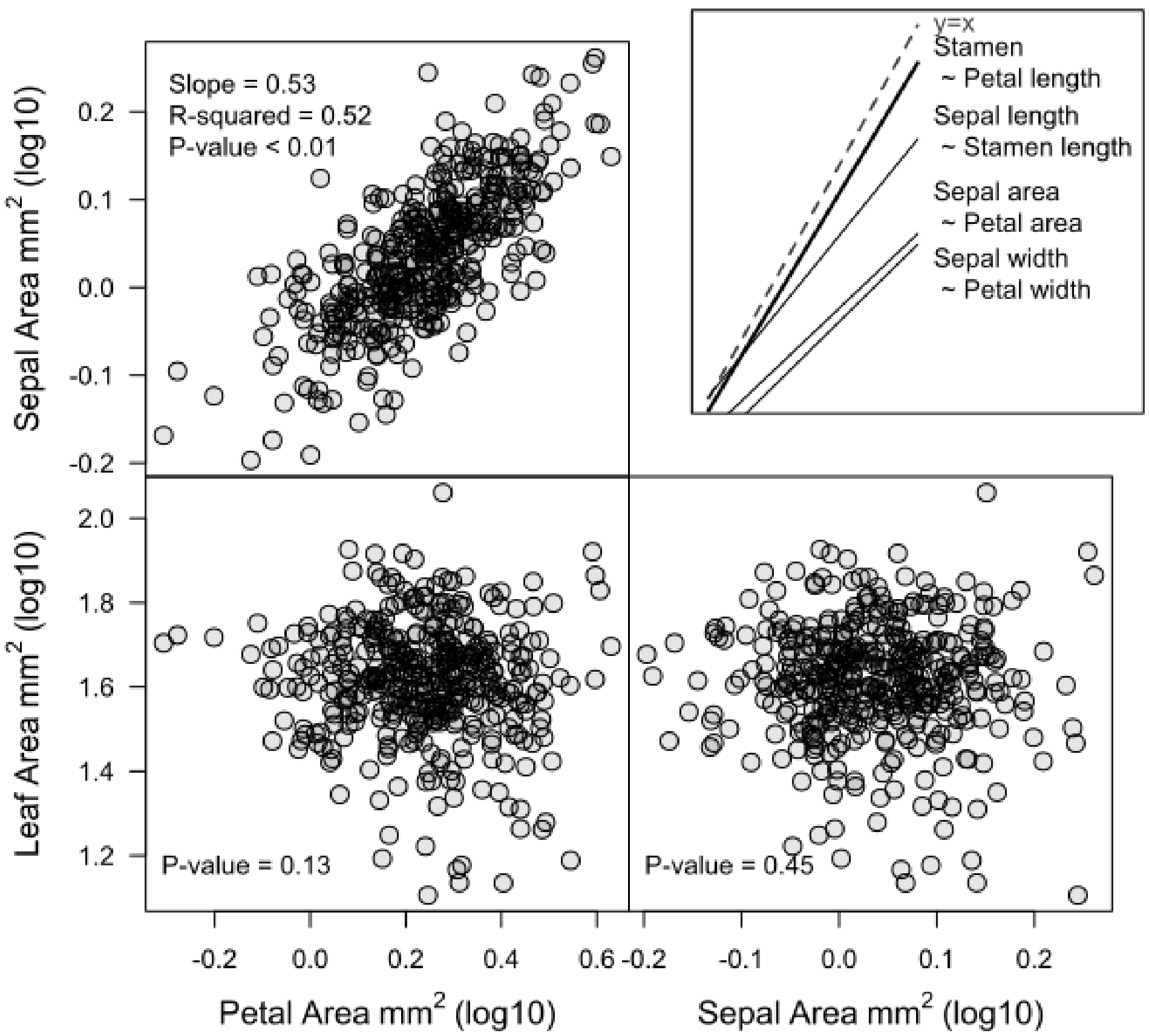

Figure S6. Pairwise relationships between organ sizes. Coefficients were obtained from standard major axis linear regressions. The top right plot illustrates the slope test for organ isometry (null hypothesis: slope = 1).

MOLECULAR ECOLOGY

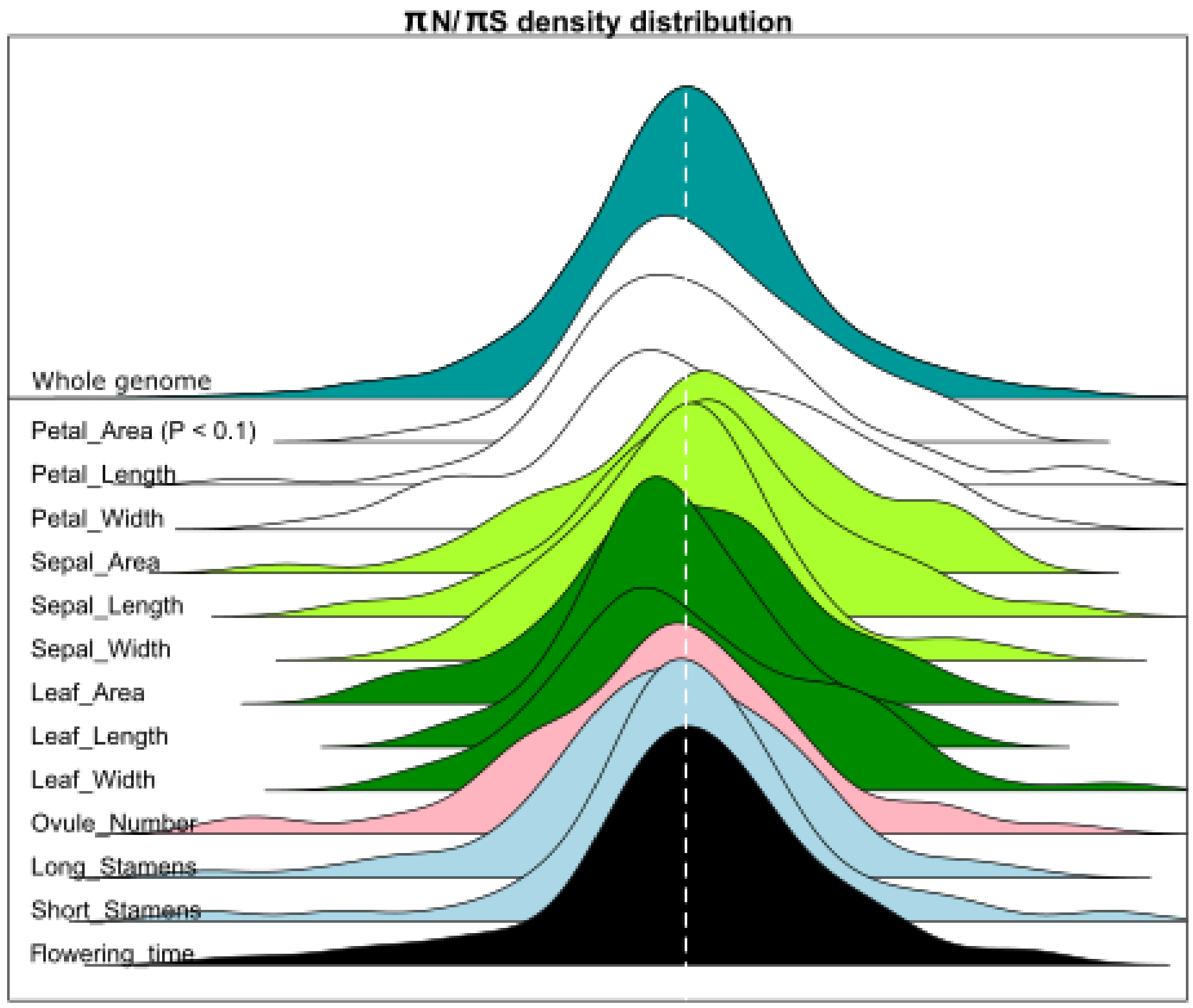

Fig. S7. ΠN/πS distribution from genes associated with leaf and flower traits, and flowering time. The dark blue distribution and its average (dashed white line) are from the genome wide computation. Trait names can be read at the left foot of the distributions. P-values indicate the significance of the mean difference between a trait and the genome wide distribution and are reported only if $P<0.1$.

MOLECULAR ECOLOGY

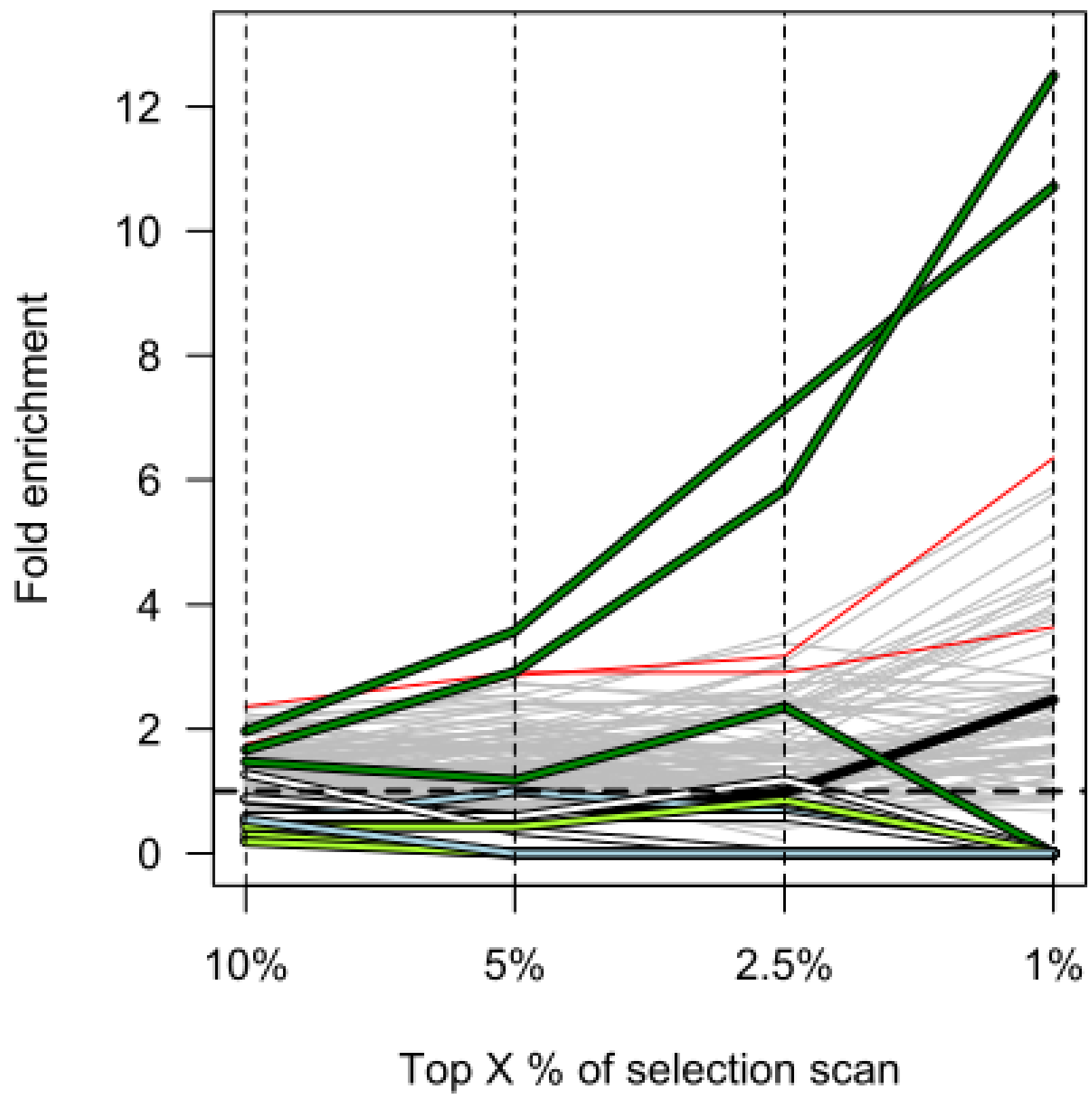

Figure S8. Fold enrichment of SNPs associated with a trait, among the most significant SNPs on a genome wide scan for selection (Extended Haplotype Homozygosity). Colors represent only the color code used throughout the manuscript; Petal, white; Sepal, light green; Leaf, dark green; Stamen, light blue; Flowering time, black. Grey lines represent the fold enrichment of SNPs detected in GWAs for all 107 phenotypes from (Atwell et al., 2010), red lines represent the enrichment detected for male and female gamete in (Tsuchimatsu et al., 2020).

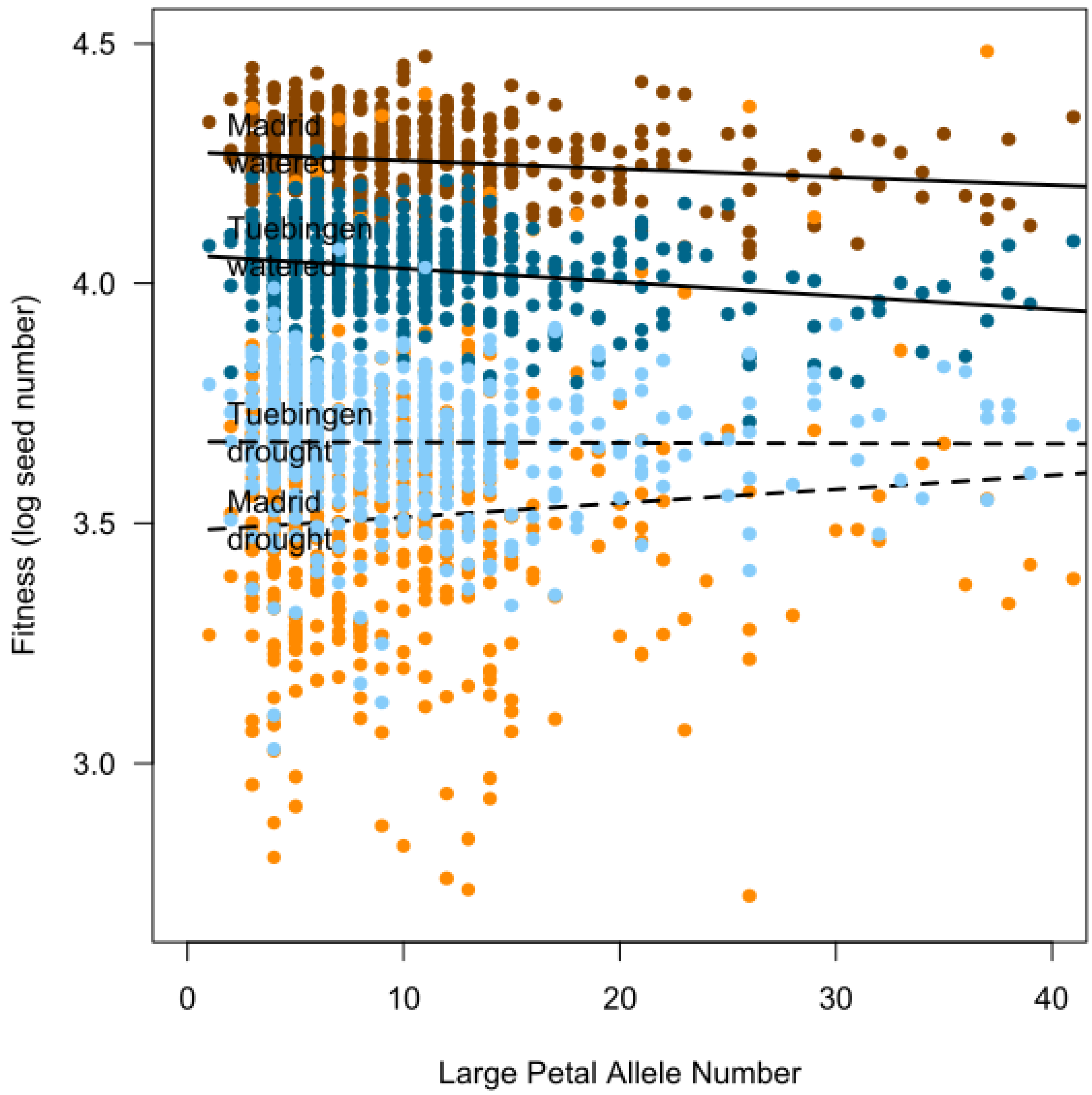

Figure S9. Fitness as a function of large petal allele number across *Arabidopsis thaliana* accessions. Fitness data were recovered from (Exposito-Alonso et al., 2019). Bold lines represent significant linear regressions; dashed lines represent non-significant regressions.

MOLECULAR ECOLOGY

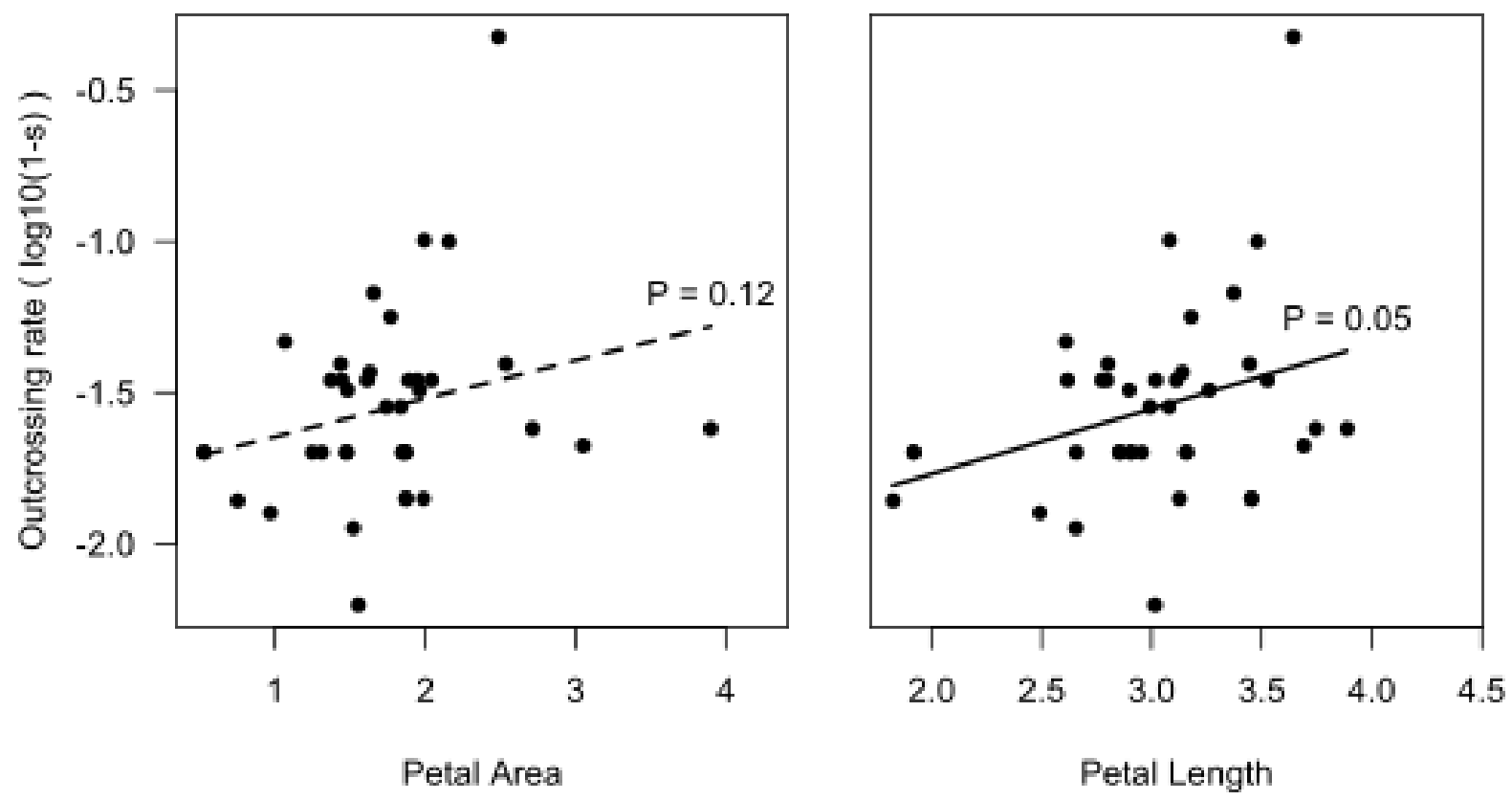

Figure S10. Outcrossing rate as a function of petal area and petal length. Each dot is a population of *Arabidopsis thaliana*; straight lines represent significant (bold) and non-significant (dashed) relationships with associated P-values (P).

References

Alonso-Blanco, C., Andrade, J., Becker, C., Bemm, F., Bergelson, J., Borgwardt, K. M., Cao, J., Chae, E., Dezwaan, T. M., Ding, W., Ecker, J. R., Exposito-Alonso, M., Farlow, A., Fitz, J., Gan, X., Grimm, D. G., Hancock, A. M., Henz, S. R., Holm, S., … Zhou, X. (2016). 1,135 Genomes Reveal the Global Pattern of Polymorphism in Arabidopsis thaliana. *Cell*, *166*(2), 481–491. https://doi.org/10.1016/j.cell.2016.05.063

Atwell, S., Huang, Y. S., Vilhjálmsson, B. J., Willems, G., Horton, M., Li, Y., Meng, D., Platt, A., Tarone, A. M., Hu, T. T., Jiang, R., Muliyati, N. W., Zhang, X., Amer, M. A., Baxter, I., Brachi, B., Chory, J., Dean, C., Debieu, M., … Nordborg, M. (2010). Genome-wide association study of 107 phenotypes in Arabidopsis thaliana inbred lines. *Nature*, *465*(7298), 627–631. https://doi.org/10.1038/nature08800

Exposito-Alonso, M., Burbano, H. A., Bossdorf, O., Nielsen, R., & Weigel, D. (2019). Natural selection on the Arabidopsis thaliana genome in present and future climates. *Nature*, 1–5. https://doi.org/10.1038/s41586-019-1520-9

Tsuchimatsu, T., Kakui, H., Yamazaki, M., Marona, C., Tsutsui, H., Hedhly, A., Meng, D., Sato, Y., Städler, T., Grossniklaus, U., Kanaoka, M. M., Lenhard, M., Nordborg, M., & Shimizu, K. K. (2020). Adaptive reduction of male gamete number in the selfing plant Arabidopsis thaliana. *Nature Communications 2020 11:1*, *11*(1), 1–9. https://doi.org/10.1038/s41467-020-16679-7
